# “It was just for us”: qualitative evaluation of an exercise intervention for African-American couples

**DOI:** 10.1186/s12889-021-10659-2

**Published:** 2021-05-01

**Authors:** Lyndsey M. Hornbuckle, Cristina S. Barroso, Amy Rauer, Chloe S. Jones, Kerri M. Winters-Stone

**Affiliations:** 1 Department of Kinesiology, Recreation, & Sport Studies, University of Tennessee, 322 HPER Building, 1914 Andy Holt Avenue, Knoxville, USA; 2 Department of Public Health, University of Tennessee, 390 HPER Building, 1914 Andy Holt Avenue, Knoxville, USA; 3 Department of Child & Family Studies, University of Tennessee, 115 Jesse Harris Building, 1215 W. Cumberland Avenue, Knoxville, USA; 4 School of Nursing and Knight Cancer Institute, Oregon Health & Science University, 3455 SW US Veterans Hospital Road, Portland, OR 97239 USA

**Keywords:** Intimate relationships/marriage, Dyads, Older adults, Resistance training, Walking, Cultural relevance

## Abstract

**Background:**

Promoting long-term exercise adherence should be a key focus for health and fitness professionals working to reduce obesity and cardiometabolic health disparities, and all-cause mortality in inactive African-American (AA) adults. Data have suggested that romantic partners can improve long-term exercise adherence and that this dyadic approach should be examined in exercise interventions. Therefore, the purpose of this study was to conduct a qualitative evaluation of a pilot exercise intervention conducted in older AA couples.

**Methods:**

Two semi-structured focus groups were utilized to compare participants’ perceptions of and experiences during the pilot intervention across two randomly assigned treatment conditions (exercising together with partner [ET; *n* = 8] versus exercising separately [ES: *n* = 6]). Participants (mean age: 64.7 ± 6.8 years) of a previous 12-week pilot exercise intervention (walking ≥3 days/week, 30 min/day plus supervised resistance training 2 days/week) were interviewed. Verbatim transcripts were coded using an open coding approach.

**Results:**

Three key themes (intervention value/benefits, intervention difficulties, and suggested improvements) emerged. Although all couples identified health and relationship benefits of the intervention, some differences surfaced within themes across the two intervention groups.

**Conclusions:**

Overall, these qualitative data suggest that couples had a positive experience while participating in the pilot study. In addition, key learning points to improve the intervention were identified including a more gradual transition to independent exercise, more flexibility training, and the incorporation of tangential education. These data will help investigators continue to develop the intervention, which is ultimately designed to promote long-term exercise adherence to reduce cardiometabolic health disparities in the AA community.

## Introduction

Exercise and physical activity (PA) researchers with an interest in addressing health inequities continue to put forth efforts to address physical inactivity in underserved and underrepresented populations. With 53% of African-American (AA) adults in the U.S. failing to meet national recommendations for both aerobic and muscle-strengthening activities compared to 46% of White adults [1], PA disparities continue to be a prominent health issue in the AA community. These disparities are problematic due to well-established data linking physical activity to reduced risk of obesity, cardiovascular disease, hypertension, Type 2 diabetes, and all-cause mortality [2]. Not surprisingly, and often linked to PA disparities, these chronic conditions and health concerns also affect the AA community at a disproportionately high rate [1, 3]. Aging AA adults are especially vulnerable as data show a high prevalence of metabolic syndrome (a cluster of cardiometabolic risk factors) in women and men 50–69 years (53 and 39%, respectively) and > 70 years of age (64 and 59%, respectively) [4], which has also been associated with physical inactivity [2].

Promoting long-term exercise adherence should thus be a central strategy for health and fitness professionals working to mitigate cardiometabolic-related health disparities. Recent studies have suggested that romantic partners can improve long-term exercise adherence and that this dyadic approach to exercise promotion should be examined in interventions [5, 6]. In addition, recent couples-based interventions in our laboratory and another support the engagement of romantic partner dyads as an effective strategy to increase physical activity and improve selected cardiometabolic risk factors [7, 8]. Both projects also showed high adherence to both the studies themselves and their protocols, which may speak to the potential for significant long-term health benefits when engaging romantic partner dyads. Supporting this dyadic focus on exercise are theories of social support and control that indicate that helping partners become more effective at both providing and receiving support to and from each other has long-term benefits, particularly as couples begin to encounter health challenges later in life [9–11]. As cultural relevance is also a recommended component that can promote acceptance and success in PA and exercise interventions [12], combining multiple levels of cultural relevance with a dyadic approach [13] may provide the novel methodology needed to have a long-term effect on exercise adherence in AA adults.

Focus groups are valuable qualitative tools that can assist investigators in the development, assessment, and tailoring of various components of PA interventions [14]. In addition to utilizing focus groups pre-intervention, several studies have shown it advantageous to use post-intervention focus groups to qualitatively evaluate the effectiveness and acceptability of a program from the participants’ perspective [15–18]. Research has shown post-intervention focus groups can assist in refining the procedures of an intervention, identifying obstacles from the participants’ perspective, and inviting suggestions for programmatic improvements [14, 15]. These data can be utilized to improve upon any weaknesses of an existing intervention study in order to inform a future iteration of that protocol.

Therefore, the purpose of the current study was to utilize focus groups to qualitatively evaluate participants’ perceptions of the pilot implementation of a 12-week exercise intervention conducted in older AA couples. The pilot intervention examined two study conditions where couples either exercised together (ET; experimental treatment) or separately (ES; active control). These conditions were tested based upon research that shows while having a health-conscious partner may improve one’s health habits [19], health benefits may be enhanced when partners work cooperatively on their health [20]. The current focus group study contributes to the investigators’ ongoing efforts to improve upon the pilot exercise intervention in order to conduct a similar intervention study on a larger scale. As such, these data will be used to inform the continued development of the exercise intervention in AA couples.

## Methods

### Summary of pilot exercise intervention study

Building upon previous couples-based intervention work in husbands and wives [6], the interdisciplinary (exercise science and family science), community-engaged pilot exercise intervention study recruited AA male-female couples who were married and/or cohabiting. Participants were recruited in the greater Knoxville, TN area via radio and newspaper advertisements, flyer distribution, speaking engagements in groups that served AA adults, and endorsements from existing participants. Briefly, the pilot intervention study aimed to use the established relationship between partners to support PA participation. The study examined the effects of prescribed walking for exercise (≥3 days/week, ≥30 min/day) plus supervised total body resistance training (2 days/week) in two randomly assigned groups (ET versus ES, defined above in the *Introduction*). Although both groups were prescribed the walking plus resistance training program, only the ET group received education during their resistance training sessions on how to facilitate partner support and enhance receptivity to partner support. This element of the program was adapted from the educational component of the Exercising Together® program [21], an existing partnered resistance training program designed to improve teamwork in couples during exercise. The ES group did not receive an educational intervention. All participants wore a wrist-mounted activity monitor and recorded the frequency, duration, and partner status (walked with or without the partner) of their prescribed walking bouts onto log forms daily for the entire 12-week study. Measured outcomes were exercise adherence, the provision of partner support and receptivity to partner health influence, and several cardiometabolic risk factors. Additional pilot study details have been published previously [7, 22].

### Participants

We used purposeful sampling for the current focus group study. Eligible participants were all of the 10 couples (*N* = 20) who had previously entered the pilot study (described above) and whose pilot study participation was not disrupted due to the COVID-19 pandemic. This decision was made in order to more effectively evaluate the intervention under typical circumstances. Couples whose participation was interrupted by the pandemic were invited to participate in a separate qualitative evaluation of their altered version of the intervention.

The ten eligible couples (five couples in each intervention group; *n* = 10/group) were contacted via e-mail or telephone and invited to participate in the focus group study. Four couples (*n* = 8) from the ET group participated, while one failed to respond to the invitation after multiple attempts. Four couples were also represented in the ES group; however, this included both partners from only two couples (*n* = 4), as two individuals (*n* = 2) attended even though their respective partners had time conflicts. Neither partner from one ES couple attended due to a time conflict. To note, this was also the only couple who dropped out of the intervention due to one of the partner’s non-study-related injury. Focus groups occurred no more than 4 months after couples completed the 12-week study.

The lead investigator (LMH) conducted two focus group meetings – one for each intervention group. To promote a comfortable environment, both focus groups were held in the banquet room of a conveniently-located local restaurant. The focus group procedures and confidentiality were discussed with the group, then participants reviewed and signed an informed consent form. All participants received lunch and a $30 gift card from a national retailer for participation in this evaluation component of the pilot study. All study procedures were reviewed and approved by the University of Tennessee, Knoxville Institutional Review Board.

### Data collection

All participants completed a brief demographic questionnaire and were informed that this information, combined with select data collected during the pilot intervention, would be used as group descriptors. Investigators developed open-ended questions with probes based upon literature related to process evaluation [14, 23] and components of the pilot exercise intervention itself in order to assess several aspects of intervention implementation. A formative approach to the process evaluation was utilized with the goal of using the data to revise and improve the intervention for a subsequent launch on a larger scale [23]. As such, focus group guide questions concentrated on facilitators and barriers (i.e., strengths and challenges), as well as interactions with the research staff and investigators. Investigators then reviewed the questions for face validity (subjective assessment of whether the developed questions would provide the data desired). Discussion between investigators resulted in a 12- (ET group) and an 11-item (ES group) open-ended semi-structured focus group guide (Table 1). Although the context of some questions varied based upon intervention group assignment, the focus groups were run identically. Part of each meeting included a separation of female and male participants to provide individuals the opportunity to provide feedback about their experience in the pilot intervention study without the concern of censoring information due to the presence of their partner. These brief break-out sessions were facilitated by gender-matched study staff.

**Table 1:** Focus group guide

**Both Groups**	
1. What do you feel were the greatest strengths of the study? (i.e., What parts of the study should definitely be used in future couples exercise intervention studies?)	
2. What do you feel were the greatest challenges of the study? (i.e., What parts of the study should be changed in future couples exercise intervention studies?) • How would you address these challenges/make the changes discussed?	
3. Do you feel the exercise program was culturally relevant? • If you do, how so? • If not, how would you improve the cultural relevance of the couples exercise program?	
4. Tell me about your experience related to the logistical and administrative aspects of the study. This may include, but is not limited to, parking on campus, completing and submitting walking logs, wearing and syncing the [activity monitoring device], completing questionnaires, etc. • How would you change or alter any of the logistical or administrative aspects of the study?	
5. Tell me about your experience with your exercise trainer. • Please identify characteristics of your exercise trainer that you enjoyed the most. • Please identify characteristics of your exercise trainer that could be improved.	
6. Tell me about your experience working with the study investigators. • Please identify characteristics of the investigators that you enjoyed the most. • Please identify characteristics of the investigators that could be improved.	
7. How could the study team have provided you with more support during the 12-week active exercise portion of the study?	
8. How could the study team have provided you with more support when you were transitioning to independent exercise immediately after the 12-week active exercise portion of the study?	
9. We now invite you to share any additional study-related thoughts or concerns. This could be related to any aspect of the pilot intervention in which you participated, or future research in couples-based exercise programs.	
**Exercised Together Group – Gender Separated Breakout Sessions**	
10. Tell me about your experience exercising together with your partner during the study. • Please comment on how this experience relates to any previous efforts to exercise together with your partner.	
11. Will you share how exercising together during this study affected your relationship with your partner?	
12. Will you share how your relationship with your partner affected your exercise habits prior to your participation in this study? • Has it changed at all since participating?	
**Exercised Separately Group – Gender Separated Breakout Session**	
10. Will you share how participating in this exercise study affected your relationship with your partner?	
11. Will you share how your relationship with your partner affected your exercise habits prior to your participation in this study? • Has it changed at all since participating?	

As recommended by Krueger and Casey [24], two research staff trained in focus group methodology recorded observations and took notes during each focus group. The lead investigator initiated the focus groups with a description of the focus group process and the purpose of the qualitative evaluation. Both focus groups were digitally audio recorded and lasted approximately 90 min. Multiple research staff members transcribed the digital audio recordings verbatim and double-checked for accuracy. The lead investigator then conducted a final accuracy check of both transcripts compared to the audio recordings.

### Data analysis

Based on the semi-structured focus group guide, two investigators (LMH and CSB) used NVivo 12 software (QSR International; Doncaster, Australia) to analyze the qualitative data using an open coding approach based on thematic analysis [25]. The two investigators independently assigned codes to concepts, opinions, or ideas that emerged from the data [25]. The coding process was iterative and collaborative, and started with a planning meeting to review procedures related to thematic analysis and determine a timeline for the analysis. The investigators held four consensus meetings. At the first two meetings, investigators presented the codes, categories, sub-categories, and the corresponding rationale that they independently identified during their review and abstraction of the de-identified focus group transcripts. Throughout the process, the investigators independently developed provisional codebooks, which contained code names and descriptions, categories, subcategories, and examples for each. The provisional codebooks were used to discuss agreements and discrepancies between coding. During the third meeting, the investigators continued to independently review, abstract, and code both focus groups. At the final meeting, the researchers finalized their rationale and merged their provisional codebooks. The final codebook contained themes identified by both investigators.

## Results

### Participant characteristics

All participants who attended the focus groups completed the 12-week exercise intervention in its entirety. In aggregate prior to entering the intervention, participants were 64.7 ± 6.8 years of age, classified as obese (body mass index 30.4 ± 4.8 kg/m^2^), and low active based on an average of 6292 ± 1615 steps/day [26]. The majority of participants (86%) reported some college education, most had part-time or full-time employment (64%), and all reported a gross annual household income of $50,000 or greater. All participants were married. Table 2 shows descriptive characteristics of participants by group.

**Table 2:** Descriptive characteristics of participants at intervention baseline (*N* = 14)

Variables	^a^Exercised Together (***n*** = 8)	^b^Exercised Separately (n = 6)
**Age (years ± SD)**	65.1 ± 6.3 (range 58–74)	64.2 ± 8.0 (range 55–72)
**Self-Identified Gender (n)**	Female: 4	Female: 3
	Male: 4	Male: 3
**Time in Current Relationship (years ± SD)**	44.3 ± 5.5 (range 37–49)	32.5 ± 15.2 (range 15–52)
**Body Mass Index (kg/m**^**2**^ **± SD)**	31.7 ± 4.6 (range 26.0–38.5)	28.7 ± 4.8 (range 23.1–36.8)
**Physical Activity (average steps/day)**	6355 ± 1308 (range 5235–7942)	6207 ± 2091 (range 2131–7881)
**Highest Education Level (n)**	High School Graduate or GED: 1	High School Graduate or GED: 1
	Some College: 1	Some College: 0
	Associate’s Degree: 1	Associate’s Degree: 0
	Bachelor’s Degree: 4	Bachelor’s Degree: 3
	Master’s Degree: 1	Master’s Degree: 2
**Employment Status (n)**	Employed Full Time: 2	Employed Full Time: 1
	Employed Part Time: 3	Employed Part Time: 0
	Self-Employed: 0	Self-Employed: 3
	Retired: 3	Retired: 2
**Gross Annual Household Income (n)**	$50,000–$74,999: 0	$50,000–$74,999: 3
	$75,000–$99,999: 2	$75,000–$99,999: 0
	$100,000 or more: 6	$100,000 or more: 3
**Relationship/Household Status (n)**	Married, with dependents: 0	Married, with dependents: 1
	Married, no dependents: 8	Married, no dependents: 5

All participants who attended the focus groups completed the 12-week exercise intervention

^a^Four couples were represented (i.e., both partners in each couple attended the focus group)

^b^Four couples were represented (i.e., both partners in two couples attended the focus group and two couples had only one partner attend)

### Qualitative themes

Data analyses revealed three key themes: 1) value and benefits of intervention participation, 2) difficulties with intervention participation, and 3) suggestions to improve the intervention. Table 3 presents the identified themes with subsequent categories and sub-categories by focus group.

**Table 3:** Identified Themes, Categories, and Sub-Categories by Focus Group (*N* = 14)

Exercised Together (***n*** = 8)	Exercised Separately (***n*** = 6)
**Theme 1: Value & Benefits of Intervention Participation**	
Study Support	Study Support
• Accountability & structure	• Accountability & structure
• Encouragement to start a fitness program	• Encouragement to start a fitness program
• Study staff	• Study staff
Partner Interaction	Partner Interaction
• Closer relationship	• Closer relationship
• Shared experience with significant other	• Shared experience with significant other
• Competition	Health Benefits
Health Benefits	• Health awareness & appreciation
• Health awareness & appreciation	• Health outcomes
• Health outcomes	Cultural Relevance
• Nutrition awareness	• AA trainers
Cultural Relevance	• Music
• AA trainers	• Prompted exercise discussions in the AA community
• Music	
• Prompted exercise discussions in the AA community	
• AA principal investigator	
Neighborhood/Community Awareness	
Self-efficacy	
**Theme 2: Difficulties with Intervention Participation**	
Study Logistics	Study Logistics
• Device issues	• Device issues
• Walking logs	• Walking logs
• Facility	Personal Barriers
Personal Barriers	• Physical challenges
• Physical challenges	• Self-motivation to attend trainings
	Training consistency
**Theme 3: Suggestions to Improve the Intervention**	
Programming	Programming
• Flexibility training	• Flexibility training
• Longer program duration	• Longer program duration
• Education	• Education
Enhanced Transition Support	• Study results to participants
Exercise Promotion in the AA Community	• Investigator check-in on workouts
• Local community engagement	Enhanced Transition Support
• Future recruitment	

*AA* African-American

### Theme 1: Value & benefits of intervention participation

Participants in both groups were pleased overall with the intervention and reported several valued benefits of participation. In the *Study Support* category, both groups expressed that they valued the accountability and structure, encouragement to start a fitness program, and study staff. Participants confirmed that the establishment and monitoring of both a walking routine and scheduled resistance training appointments helped them adhere to the prescribed exercise. Otherwise, the participants indicated they would have been less likely to participate regularly.

ET male: *...you know we knew that every Tuesday and Thursday we HAD to be there so we made sure we was in place … but when you ain’t got nobody to hold you accountable to that … then it’s kind of like okay well … I ain’t gotta meet nobody this morning so I guess I can go on and sleep in a little bit today …*

ES male: *I think the structure for each of the exercises that we each have to do, the consistency of that was supportive for me ‘cause it gave me each day … I had a vision of what, what I had to do, how often I had to engage in it, and exercise. And I can personally see any kind of improvement that may have taken place from the time I started to the next day doing the exercises.*

Participants in both groups discussed how the pilot study helped encourage them and to prepare mentally for engagement in exercise. The participants also expressed appreciation for the interactions they had with the study staff while engaging in the exercise activities because they were courteous, supportive, and “refreshing.”

ES female: *It helped to jumpstart us as well, because we know we needed to do something, and then this just made us do … you know when it happened [they heard about the pilot study], it made us like ‘okay, this is the time to just get started’ and we needed that boost. So I appreciate the study just getting us going.*

ET female: *… you need to have a special … I guess umm, aptitude … and be willing to work with all different types of people in different situations and she [exercise trainer] definitely has you know, those characteristics.*

In the *Partner Interaction* category, participants in both groups discussed how their participation in the pilot intervention helped them to feel closer to their partner by improving relationship dynamics and/or communication. Unlike the ES group, the ET group also discussed the benefit of having regularly scheduled time and quality time together as a valued benefit of participating in the study. The following exchange between participants resonated with investigators:

ET female 1: *Just for us. ‘Cause we do a lot … well you know he’s a pastor … and with him … we do an awful lot for other people. But [the exercise study was] just for us! I liked it.*

ET female 2: *And that’s what I liked too. It was just for us.*

ET female 1: *Just for the two of you.*

Participants also mentioned how sharing the study experience with their significant other helped facilitate discussions about exercise with their partners.

ET male: *I think the greatest strength was the co-ed experience. I’m not sure I would have been as committed to this … with a group of guys … as I was having the prodding from my significant other.*

Only the ET group discussed competition between partners. Participants in the ET group reported that exercising together spurred friendly rivalry in completing the prescribed exercise activities and/or re-ignited their competitiveness.

ET female: *Yea … why waste the time if you’re not gonna put everything into it? … we would have some conversations sometimes about how I would do mine [resistance training exercises] so much faster. So he said ‘you don’t put enough into it.’ So we get in a little disagreement about that. But still at the end of the day we were back to where we needed to be. On the same level you know. But it’s always, with us, a little bit of competition.*

Both groups recognized that involvement in the pilot study evoked a deeper concern for overall health and well-being (*Health Benefits* category). More specifically, this included a new awareness of physical health and functionality, as well as an awareness of their own vitality compared to their peers. Participants also discussed how their health and/or fitness improved because of their participation in the intervention. Only the ET group discussed developing more awareness of their dietary habits.

ET male: *...you become aware of the folks around you who appear to be in poor physical shape and you start scratching and wondering … did I look like that? Or is that where I was headed? So it does sensitize you to the issue of physical fitness. It’s important.*

ET male: *We also started … uhh … discussing our nutrition a little bit more together because of the study. So we were a little bit more aware of the carbs and the sugar and all those things, and I think that the study is what caused us to think more about those things.*

Both groups valued aspects of *Cultural Relevance* that were present in the intervention. Both groups commented on the benefits of working with AA trainers, including comfortable small talk and their desire not to disappoint the trainer. Only ET participants stated that they wanted to participate in the intervention study because the lead investigator was AA. Participants in both groups also discussed their preference to exercise to music popular in the AA community and appreciated the fact that the study staff played the participants’ choice of music as a motivator and comfort measure while exercising in the laboratory. Finally, both groups mentioned examples of how their study participation prompted discussions about exercise when conversing with others in the AA community, which some thought may help bring awareness to an increased need for AAs to become physically active.

ET male: … *I think having an African-American trainer made it a lot easier. And, in a way I suppose I felt like we were letting her down if we didn’t give her our best effort. Not sure I would have felt that way if it had been a trainer of a different race.*

ES male: *My trainer played good Black music so that made me get ready to go! (group laughter).*

ET male: *Well I feel like, number one did … sort of stimulate us in the community as African-American couples to get more active in working out and I think if we start really … promoting it to others to really get them involved, I think it is going to help our community as a whole …*

Only the ET participants discussed new knowledge about and experiences within their *Neighborhood and Community* and highlighted improvements in their exercise *Self-Efficacy*.

ET female: *… so we walked just about all over [county name]. And we learned a lot … this is where this street goes, this is where we are. So it was...it was really good to be able to do that together. And we would see people that we knew.*

ET female: *It gave me a different mindset. I mean … ‘cause like I have never … I’m talking about have NEVER lifted weights. … I mean I have walked … but weights … this was BRAND new to me. But I liked it!*

ET female: *I walked up hills. You know like … steep hills … I just walked all the way around them … steep hills.*

### Theme 2: Difficulties with intervention participation

Participants also noted challenging aspects of their experience while participating in the intervention. In the *Study Logistics* category, several participants expressed frustration when their activity monitoring devices (all newly purchased for the pilot study) malfunctioned before they completed the 12-week program. Participants in both groups reported issues with device synchronization and devices not holding a charge. Most participants also found completion of the daily walking record and its weekly submission to be cumbersome. One ET participant discussed undesirable aspects of the restrooms at the exercise laboratory.

ET female: *Yeah, it [electronic tracking device] just stopped. Stopped working.*

ES male: *I certainly didn’t like that log, but I knew that it was required to study so it didn’t bother me. I mean, I knew that that information was vital to what we were doing. I didn’t like doing it and I would have to make up a couple of days at the time.*

As for *Personal Barriers*, participants in both groups described relatively minor physical challenges (e.g., chronic back pain, previous injuries) as an obstacle to exercise engagement.

ET male: *… when I first started I was having, you know my back flared up and I went through it [the exercise intervention] … it got better.*

The groups differed in this category as the ES group discussed self-motivation to attend trainings and inconsistent training instructions as perceived challenges during their participation.

ES male: *It was real easy to make an excuse on why I didn’t want to exercise that day.*

Finally, one ES participant brought to light his experience with *Training Consistency* on occasions when a substitute trainer needed to step in for his regular trainer. Namely, this participant discussed differences in the exercise intensity (i.e., rest time between sets) between trainers and the need to be mentally prepared to work harder for one compared to another.

ES male: *You know, the goals of the trainer should be the same. Here’s what we’re trying to accomplish. Here’s how we’re going to do it.*

### Theme 3: Suggestions to improve the intervention

Participants in both focus groups were greatly invested in the pilot intervention and therefore, were candid when making suggestions on how to improve it. Categories identified in both groups under this theme were *Programming*, including calls for more flexibility training and a longer program duration, and *Enhanced Transition Support* as participants graduated from supervised training to independent exercise at their respective fitness facilities.

ET male: *Print out several stretches you can do at home, sit off the edge of your seat, and do a lot of them.*

ET female: *… I think longer, definitely longer than 12 weeks [referring to the duration of the intervention]. Maybe … about 6 months really to get you into that mode … because … exercise is just important that you have to do for the rest of your life...as we get older …*

ES female: *As far as, you know, when we transition to the club … help us. … I mean, you weren’t able to go through the club with us on the machines and stuff, but that would have been nice too.*

Group differences under *Programming* included the ET group’s suggestion to incorporate different educational components into the intervention (e.g., nutrition, study background/methods). The ES group suggested more involvement in the day-to-day exercise activities from the lead investigator, including more frequent check-ins and more research team meetings about the participants’ progress. The ES participants also wanted to learn more about the results of the research study (e.g., participant aggregate progress, what worked to help participants become more physically active, and the publication of the study results).

ES female: *… maybe coming in more than once or so, or just dropping in checking us out.*

*Exercise Promotion in the AA Community* was a category that only surfaced in the ET group where participants expressed a desire to help other AA community members engage in exercise and presented innovative ideas to recruit future intervention participants. These suggestions relate to community participation, development, and empowerment. In essence, all of these suggestions revealed a deep commitment to the success of the intervention study and opportunities to increase PA in the local AA community, which could have a positive influence on health.

ET female: *… well we have a center, a Martin Luther King Center that we could talk with the person that runs it. And so why not put something like this [the pilot study exercise intervention] in play? Because mostly African-Americans … use it, so I was just wondering if that might be a way to reach out to others.*

ET male: *I think at the end of the program, if you gave us some talking points, and we went to individuals that we think would be candidates, and we would use your talking points to sell the program.*

## Discussion

To our knowledge, the pilot exercise intervention study referenced and evaluated in this paper [7] is the first exercise intervention to use romantic partner to promote exercise and reduce cardiometabolic risk in older AA adults. As such, the data collected during this qualitative evaluation are also novel and more importantly, they are critical in providing a comprehensive assessment of the pilot exercise intervention study being examined. Consideration of the participants’ experiences during and following the intervention will be integral as investigators work to improve and develop the intervention in the future.

### Intervention Value & Benefits

Both intervention groups identified the accountability and structure, encouragement to start a fitness program, and the shared experience with their significant other as key strengths of the pilot study. Research examining PA barriers in AA women and men has cited lack of time, lack of motivation, and lack of a PA partner/social support as issues [27, 28]. The current participants’ specific examples of how study participation required them to prioritize exercise over a myriad of other daily activities, even when they were not intrinsically motivated to do so, is important as it addresses these barriers. Cultural relevance and study staff were strengths identified by participants that addressed additional cited barriers to PA including a lack of physically active role models and lack of knowledge about how to use exercise equipment [28]. Based on the responses of the focus group participants, there appeared to be a level of overlap between the cultural relevance and the study staff categories, as the majority of the study staff and all of the exercise trainers were AA. These findings will inform the refinement of this intervention, in particular, the importance of having cultural relevance woven throughout the intervention and the use of AA study staff. This aligns with current literature that speaks to the importance of cultural relevance in research [12, 13].

One less expected theme to emerge was that the ET group identified competition between the partners as a study strength. The authors speculate that ET was the only group that mentioned competition between partners given the inherent dynamic that included direct and intentional interpersonal interactions during exercise. This finding suggests that for self-proclaimed competitive personality types, an underlying sense of rivalry may be elicited despite education during their supervised resistance training sessions on how to encourage each other and work as a team. That said, the competitive dynamic noted by some appeared to be affable overall and therefore, did not seem to negate the positive relational benefits of the study. Future research may wish to examine the effect of a competitive relationship and exercise adherence between partners. What may be key is whether spouses share a similar competitive approach to exercise, as discrepant views in this regard may diminish the benefits of joint exercise [29].

Although not the focus of the current evaluation, investigators note that participants provided limited qualitative impact evaluation data related to the study’s influence on the relationship dynamics. Both groups noted feelings of having achieved a closer relationship with their partner after participating in the intervention. However, the investigators found it interesting that the dynamic between the two intervention groups appeared to influence participants’ perceptions of the intervention’s benefits. The ET group acknowledged the study as both exercise and an opportunity to regularly connect with their partner. This connection was described both as time spent together completing the prescribed exercise and time spent participating in typical activities of daily living. It was apparent that this time spent together was valued and special to the ET group, particularly the women. All four of these women identified exercising together during the intervention as one of the few activities, if not the only one, that the couple did together just for themselves. The finding that women, in particular, noted the relationship benefits of exercising together is consistent with previous research [30] that examined the relational outcomes of couples who participated in the Exercising Together® program [21, 31]. Partners in this randomized controlled exercise trial, comprised of males recovering from prostate cancer and their female spouses, appeared to derive different benefits from exercising together. Only the females in the study showed improved relational functioning (e.g., increased affection) as a result of exercising together, whereas there were no changes for the male partners. The authors concluded that women may benefit from collaboration and be particularly attuned to their relationship functioning. Thus, women in the Exercising Together® study and the current one, may have appreciated the intervention as an opportunity to dedicate time and energy to renewing their bond with their spouse.

Participants in the ET group also disclosed feelings of long-term marriages having “died down,” but that the exercise study introduced a new and positive element into the relationship. This line of conversation did not surface in the ES group. In contrast to the ET group, the ES group appeared to view the study largely as an exercise intervention and mainly focused on physical health benefits, despite having referenced some tangential relationship benefits. Although it is plausible that the longer relationship duration of the ET group compared to the ES group (44.3 vs. 32.5 years, respectively) may explain why the former was reporting more of a relationship lull, recent longitudinal work on couples suggests that there is little evidence of deterioration in marital quality over the marital life course [32]. Thus, we suggest that it is unlikely that there were significant a priori differences in the relationships of couples assigned to the two groups, but instead speculate that the experience of exercising together may have drawn those partners’ attention to their relationship in a reflective and seemingly beneficial manner.

The ET group also noted opportunities to explore their neighborhoods and the surrounding community, a heightened awareness of nutrition habits, and self-efficacy as benefits of participation in the pilot study. Investigators reference the discussion above regarding the ES group viewing the pilot study with a more straight-forward lens (i.e., exercise intervention) to offer speculation as to why these benefits did not surface in the ES group. It may be that in being directed to focus not only on their own progress but their partner’s as well, the ET participants expanded their focus beyond exercise to think about health and well-being more holistically (e.g., nutrition) and more collectively (e.g., spouse, the community).

### Intervention difficulties

As wearing a PA monitoring device has been linked to improvements in PA [33] and self-monitoring has been clearly shown to promote behavior awareness (and likely to contribute to successful behavior change) [34], investigators are somewhat discouraged that the two monitoring strategies employed by the pilot intervention were viewed by participants as challenging. The functional issues experienced when using the consumer-grade, objective activity monitoring devices chosen for the study were unexpected, but still provoked a negative study-related experience for participants and staff. The device was chosen for its wear ease (wrist-mounted) and validity [35]. However, most participants recommended that model and/or the manufacturer not be used again. It is noteworthy that ES participants reported their desire to help the lead investigator’s research agenda as a motivator to record their walking activities, even though they found it to be a cumbersome study-related task. This demonstrates that community buy-in includes PA research conducted by AA researchers. Investigators are considering more convenient ways to acquire reports of daily walking that could replace the written log in the future.

Although relatively minor physical challenges were noted in both groups and are not uncommon in aging individuals, two ES participants perceived the exercise activities as something that they needed to push themselves to do. Even so, those participants and others expressed gratitude to be able to rely on the structure of the study to facilitate exercise participation and eliminate excuses. Diminished motivation did not emerge in the ET group. These accounts may be supported by data that link exercise enjoyment and group dynamics (between the participant, the exercise leader, and other participants), showing that enjoyment and intention for continued participation is optimized in the presence of a supportive group environment that is directed by a positive, supportive leader [36]. The ES group only interacted one-on-one with their exercise trainer during resistance training sessions and were prescribed to complete their walking alone, whereas ET always had a partner with them. That said, it could be argued that social support was also provided to the ES participants given partners entered the pilot study together. The current investigators will continue to explore how different configurations of dyadic engagement can affect exercise participation and adherence.

### Suggestions for improvement

The suggested intervention improvement that was clearly the most urgent to participants was the call for more support when transitioning from the study to independent exercise. One participant candidly expressed feeling dropped “like a hot potato.” Although multiple mechanisms were in place that aimed to assist participants in their transition (e.g., 12 weeks of guidance to build exercise knowledge and confidence, paid commercial fitness facility memberships that provided cost-free small group classes and personal training sessions, keeping the study-distributed activity monitors), participants would have likely benefitted from a more gradual/guided change to their new exercise venues and more pointed discussions about transitioning to independent regular exercise. A common sentiment among participants was the desire to be held accountable for exercising after the 12-week program ended. Investigators agreed with participants that this could be achieved relatively easily by sending texts or e-mail reminders to exercise, and/or having study staff continue to remotely monitor activity through their activity monitors. Evidence related to exercise interventions in older adults suggests that these and other strategies can be successful in enhancing exercise sustainability, however more long-term research is needed in this area [37]. Additionally, participants expressed a desire to receive more flexibility training as a part of the study. As it stood, participants completed total body stretching with the assistance of their exercise trainer (ES) or their exercise trainer and their partner (ET) for 5–10 min immediately following their resistance training sessions. They were also encouraged to stretch independently after their walks, although no formal accountability was employed. As flexibility training is unquestionably beneficial to maintain range of motion as individuals age [38], investigators will prioritize more intense flexibility training during supervised sessions and accountability for home-based flexibility training in future studies in this population.

Community engagement and education were also prominent suggestions for program improvement posed by the ET group, yet were not mentioned in the ES group. Again, this may link to the ES participants’ more narrow perception of the pilot study solely as an exercise intervention, versus a more holistic perception of the study experience that seemed to persist in the ET group. As investigators developed the study using a community-engaged approach by consulting with a community advisory board [22], participant-generated ideas to broaden the local reach of the intervention are instrumental and highly valued to help maintain the intervention’s community ties. In addition, discussions about adapting the intervention to take place in the community (versus the laboratory gym) occurred in the ET group. Participants also expressed interest in additional background knowledge about the study methods as a means to better motivate themselves to comply with study procedures, namely, completing and submitting the walking logs. Nutrition education was also requested in terms of making healthy choices in general, as well as when and what types of foods to eat to fuel workouts. The investigators note that the addition of an educational component (outside of the ET group’s team-building education during workouts) was also suggested by our community advisory board, yet could not be executed in the pilot intervention study due to limited staff and resources.

### Limitations

The investigators acknowledge this study had limitations. First, given the overarching goal of seeking information about a specific pilot intervention study, the sampling strategy that targeted the 10 couples who entered the pilot study presented an inherent drawback of a small target participant pool of 20 potential participants (i.e., 10 participants per intervention arm). Investigators intended to invite all participants who entered the study to participate in subsequent focus groups; however, all couples who entered the pilot study after those invited to the current focus groups had their pilot study participation disrupted due to the COVID-19 pandemic. Consequently, investigators conducted only one focus group per intervention arm and the sample size was small. This hindered investigators’ ability to make comparisons within each intervention group and could have affected the results obtained. Investigators also noted that the ES group was less represented than the ET group. Even so, investigators were satisfied that saturation was achieved as similar themes surfaced between the two groups.

The investigators also recognize that more reserved participants may be hesitant to contribute to a focus group discussion. As such, these participants may not fully engage in the discussion and allow others to control the conversation. To minimize this, the focus group moderator monitored participant contributions and involved all participants. That said, the moderator observed that the small sample size appeared to organically enable a more intimate discussion during which all individuals willingly participated. Finally, investigators’ experiences can bias the analysis and interpretation of qualitative data. To prevent potential bias, notes taken at the focus groups by trained note takers supplemented the transcriptions ensured the context and accuracy of the data. Additionally, holding multiple consensus meetings with study staff that both attended and did not attend the focus group meetings allowed for the confirmation of data accuracy and analysis. Examination of data accuracy and confirmation of data interpretation helped to establish the credibility of the data and the study’s trustworthiness [39]. Although these data are not generalizable to all AAs or older adults, these findings may be transferable to comparable older AA adults living in the Southeastern region of the U.S. Despite these limitations, this study is valuable as it begins to establish a line of research that examines a novel, dyadic approach to exercise promotion in the AA community.

## Conclusions & future direction

Overall, our results suggest that the couples had a largely positive experience in the study. Investigators were pleased that several participants mentioned their desire to “come back” to or repeat the study, and that many strengths of the program were recognized. Key learning points within the value and benefits theme included: 1) the importance of study staff selection to promote a generally positive study experience (e.g., patient and encouraging personality traits) and promote cultural relevance (e.g., an expressed comfort with AA trainers); 2) perceived value of exercise for personal long-term health and the health of the AA community; and 3) perceived relationship benefits that were highly valued. Additionally, participants identified some programmatic challenges and provided insightful suggestions to improve upon the pilot study that will be essential as investigators work to develop future versions of the intervention. The difficulties experienced by participants have been noted and will be addressed moving forward. Investigators consider the following key learning points perhaps most critical for improving the intervention: 1) a more gradual, supportive transition to independent exercise; 2) more frequent flexibility training to support a well-rounded physical training program; and 3) the incorporation of tangential education (e.g., rationale for study methods, proper nutrition to support new exercise participation, etc.). Ultimately, the current data will be used to inform research strategies to promote long-term exercise adherence in AA adults moving forward, in an effort to make an impact on cardiometabolic health disparities in this population.

## Data Availability

Data are not available to the public. However, data are available to the publisher upon reasonable request for any verification purposes. Any data sharing must first be approved by the University of Tennessee, Knoxville Institutional Review Board.

## Abbreviations

AA: African-American
ES: Exercised separately
ET: Exercised together
PA: Physical activity
